# Itraconazole and posaconazole, inhibitors of NPC1 sterol transport, act as pharmacological chaperones after washout

**DOI:** 10.1016/j.jbc.2025.110370

**Published:** 2025-06-16

**Authors:** Weixiang A. Wang, Cheng-I.J. Ma, Noah Steinfeld, Frederick R. Maxfield

**Affiliations:** Department of Biochemistry, Weill Cornell Medicine, New York, New York, USA

**Keywords:** lysosomal storage disease, NPC1, pharmacological chaperones, cholesterol

## Abstract

Niemann-Pick type C (NPC) disease is a rare lysosomal storage disorder primarily caused by mutations in the NPC Cholesterol Transporter 1 (*NPC1*) gene, resulting in cholesterol and lipid accumulation in late endosomes and lysosomes. While several therapeutic drugs show promise in reducing cholesterol accumulation, none of the current treatments are highly effective. Itraconazole and posaconazole, widely used antifungal drugs, have been shown to stabilize misfolded NPC1 proteins, enabling their escape from endoplasmic reticulum-associated degradation. This chaperone-like property makes them attractive candidates for testing chaperones as possible treatments for NPC disease, but both drugs also inhibit NPC1 function. In this study, we employed a washout approach to reverse the inhibitory effects of these drugs, leveraging the fact that wild-type NPC1 proteins have a half-life of about 42 h. Treating *NPC1*^*I1061T/I1061T*^ human fibroblasts with itraconazole or posaconazole for 72 h, followed by 24 to 48 h of washout, we observed a significant reduction in lysosomal cholesterol accumulation. A modest rebound was observed 72 h after drug removal, likely due to protein turnover. We also tested a repeated pulsed exposure treatment, in which short drug treatments were followed by extended washout periods. This strategy preserved the functional benefit of NPC1 stabilization while minimizing inhibitory effects. These findings indicate that a washout strategy can enhance the functional benefits of pharmacological chaperones, offering a potential future therapeutic approach for NPC disease.

Niemann-Pick type C (NPC) disease is a rare, autosomal recessive lysosomal storage disorder characterized by the accumulation of unesterified cholesterol and other lipids in late endosomes and lysosomes (LE/Ly) (1). The disease is caused by mutations in the *NPC1* or *NPC2* genes, with approximately 95% of cases attributed to mutations in *NPC1* (2). *NPC1* encodes a multispan transmembrane protein essential for intracellular cholesterol transport out of lysosomes. Among the over 400 identified mutations, the I1061T missense mutation is one of the most common (3, 4). Missense mutations often result in misfolded NPC1 proteins that undergo endoplasmic reticulum-associated degradation, thereby blocking its cellular function and leading to impaired cholesterol trafficking (5).

Several therapeutic strategies have been investigated for NPC disease. One promising approach involves 2-hydroxypropyl-β-cyclodextrin (HPβCD), a compound that solubilizes cholesterol and facilitates its lysosomal clearance (6). In preclinical models, HPβCD treatment has been shown to reduce unesterified cholesterol and glycolipid storage, delay clinical onset, and prolong survival (7, 8, 9). However, its inability to cross the blood-brain barrier presents a significant challenge for broader clinical application (9).

Alternative strategies focus on enhancing the folding and stability of mutant NPC1 proteins. Arimoclomol, recently approved by the U.S. Food and Drug Administration for NPC disease, has been reported to have beneficial effects, perhaps by stabilizing NPC1 (10). However, direct tests of improvement in folding mutant NPC1^I1061T^ in human fibroblasts did not show any improvement in folding and transport of the mutant proteins in cells treated with Arimoclomol (11). Histone deacetylase inhibitors have demonstrated the ability to improve the folding and trafficking of the majority of NPC1mutants by modulating chaperone activity (12, 13, 14). Oxysterols, including 25-hydroxycholesterol and mo56-hydroxycholesterol, act as pharmacological chaperones that demonstrate potential to stabilize mutant NPC1 proteins and partially restore cholesterol homeostasis (15, 16). However, these compounds are not specific to NPC1 and exhibit significant off-target effects. Thus, there is a critical need to identify new therapeutic approaches that can restore NPC1 function.

Itraconazole and posaconazole, commonly used as antifungal agents (17, 18), have been identified as potential pharmacological chaperones that can facilitate the proper folding of NPC1 (16). However, previous studies also showed that these two drugs act as inhibitors of NPC1 function (19), which introduces a paradoxical effect; while they may promote the folding and trafficking of NPC1, they also inhibit its cholesterol transport activity.

Given that the half-life of wild-type NPC1 proteins is approximately 42 h (5, 12), we hypothesized that a drug washout would allow the correctly folded and trafficked NPC1 proteins to function in the absence of continued inhibition by the two drugs. In this study, we investigated the effects of these drugs on lysosomal cholesterol accumulation in both normal human fibroblasts, *NPC1*^*I1061T/I1061T*^ human fibroblasts, and *NPC1*^*N1156S/R1186H*^ human fibroblasts. We tested our hypothesis using two distinct treatments. In the first treatment, cells were continuously exposed to the drugs for 72 h, followed by an extended washout period of up to 72 h. In the second treatment, cells underwent repeated pulse dosing over 4 days, with each day consisting of 6 h of drug exposure followed by an 18-h washout period.

Our findings provide insights into the dual effects of itraconazole and posaconazole on NPC1 function and highlight the potential for optimizing new NPC1 inhibitors as pharmacological chaperones for the treatment of NPC disease.

## Results

### Itraconazole and posaconazole increase lysosomal cholesterol accumulation in normal human fibroblasts

**Figure 1:**
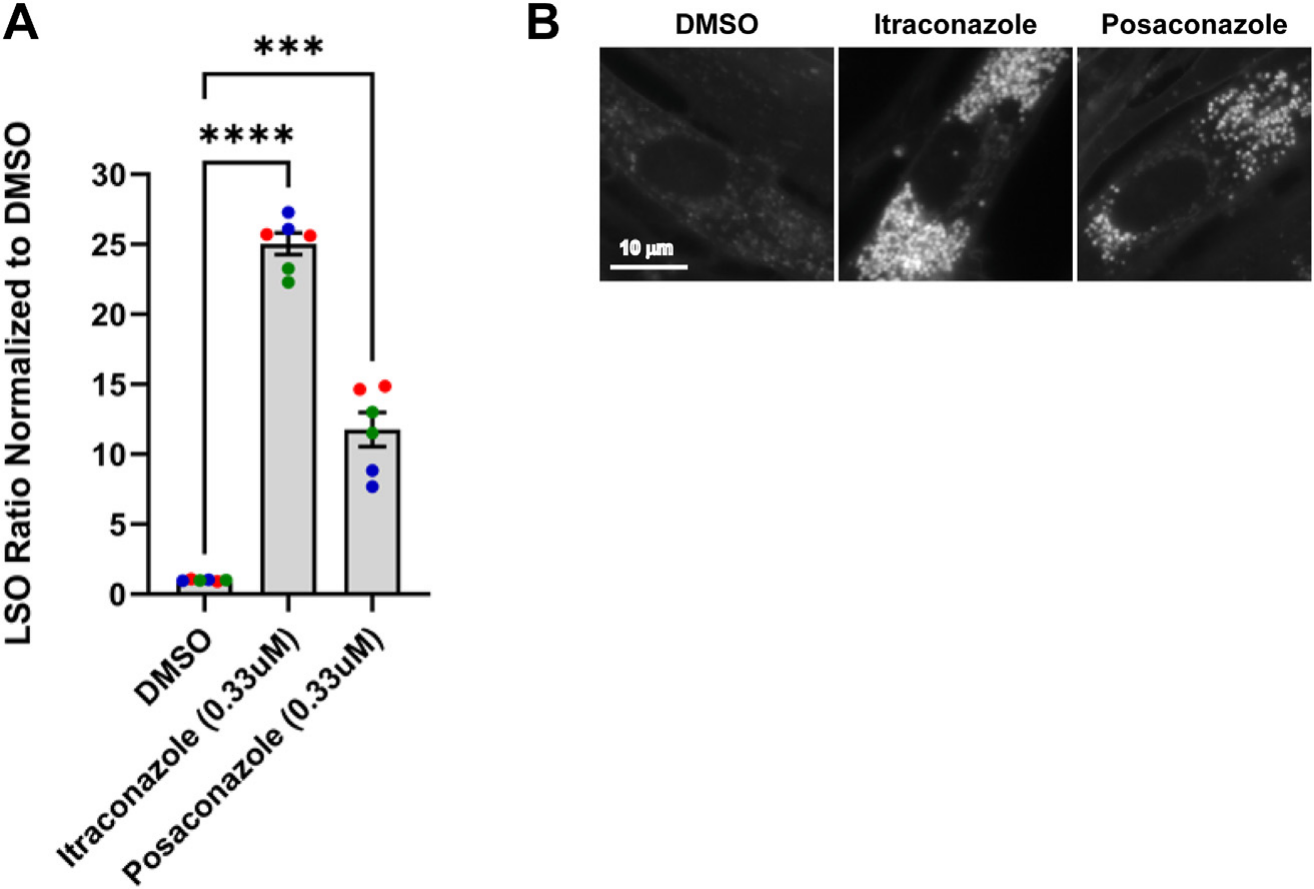
**Effect of itraconazole and posaconazole on normal human fibroblasts**. *A*, normal human fibroblasts were treated with itraconazole or posaconazole at a final concentration of 0.33 μM for 72 h. Cells were fixed, then stained with filipin to visualize unesterified cholesterol and Draq5 to stain nuclei for cell number. The LSO per cell values were measured to determine the relative amount of stored cholesterol in LE/Ly. Data were from 3 independent experiments labeled with different colors, and each data point was obtained by using nine field of views in one well. Each field of view contains about 200 to 400 cells. LSO values in DMSO-treated cells were measured in each experiment for normalization. The LSO value of one indicates the value in DMSO controls. Error bars show standard deviation (SD). *B*, representative fluorescence images of normal human fibroblasts treated with DMSO (*left*) itraconazole (*middle*) and posaconazole (*right*) and stained with filipin (*gray*). Scale bar = 10 μm. ∗∗∗, *p* ≤ 0.001; ∗∗∗∗, *p* ≤ 0.0001.

Treatment with itraconazole or posaconazole led to significant lysosomal cholesterol accumulation compared to DMSO in normal human fibroblasts (Fig. 1, *A* and *B*), confirming that both drugs can cause lysosomal storage of cholesterol, presumably by inhibiting NPC1 function (19). To quantify cholesterol storage, cells were stained with filipin, a fluorescent dye that binds free cholesterol. Specifically, we measured the fluorescence intensity in bright foci (lysosomes) divided by the cell area as determined using a low threshold for filipin fluorescence, which labels the whole cell (11). This ratio, referred to as the LSO (lysosomal storage organelle) ratio, was used as a quantitative metric of lysosomal cholesterol accumulation.

### Time and dose-dependent effects of itraconazole and posaconazole on *NPC1*^*I1061T/T/I1061T*^ human fibroblasts

**Figure 2:**
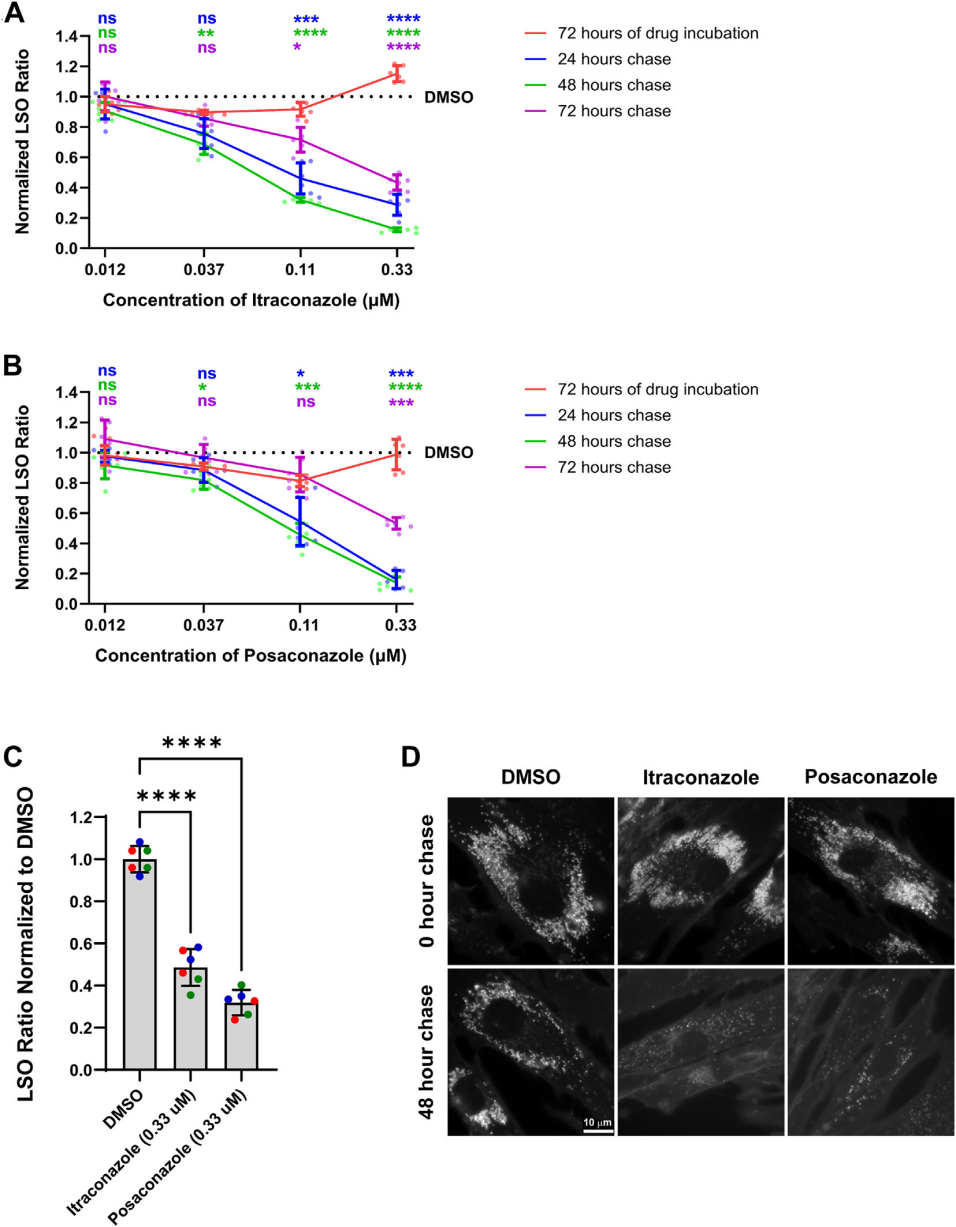
**Time and dose-dependent effects of itraconazole and posaconazole on *NPC1*^*I1061T/I1061T*^ human fibroblasts**. *NPC1*^*I1061T/I1061T*^ human fibroblasts were treated with itraconazole (*A*) or posaconazole (*B*) at 4 different concentrations ranging from 12 nM to 0.33 μM for 72 h, and chased for an additional 0, 24, 48, or 72 h without drugs. At the end of each time point, cells were fixed and stained with filipin and Draq5. The LSO values per cell were measured to determine the relative amount of stored cholesterol in LE/Ly. Data were from 3 independent experiments, and each data point was obtained using nine fields in one well for each replicate. Each field contains about 200 to 600 cells. LSO values in DMSO-treated cells were measured in each experiment for normalization and shown as a dotted line. The value of one indicates DMSO controls. Different colors were assigned to represent different time points. Error bars show SD. Statistical analysis was performed using One-Way ANOVA with Dunnett’s T3 multiple comparisons test: ∗, *p* ≤ 0.05; ∗∗, *p* ≤ 0.01; ∗∗∗, *p* ≤ 0.001; ∗∗∗∗, *p* ≤ 0.0001; ns, not significant. *C*, *NPC1*^*I1061T/I1061T*^ human fibroblasts were treated with itraconazole or posaconazole at a final concentration of 0.33 μM for 6 h, followed by an 18-h washout in fresh growth medium (MEM supplemented with 5% FBS). This pulse dosing cycle was repeated daily for 4 days. After treatment, cells were fixed and then stained with filipin to visualize unesterified cholesterol and NucSpot 650/665 to label nuclei for cell number. The LSO per cell values were measured to determine the relative amount of stored cholesterol in LE/Ly. Data were from 3 independent experiments labeled with 3 different colors, and each data point was obtained by using nine field of view in one well. Each field of view contains about 50 to 250 cells. LSO values in DMSO-treated cells were measured in each experiment for normalization. The LSO value of one indicates the value in DMSO controls. Error bars show SD. Statistical analysis was performed using One-Way ANOVA with Dunnett’s T3 multiple comparisons test: ∗∗∗∗, *p* ≤ 0.0001. *D*, representative fluorescence images of *NPC1*^*I1061T/I1061T*^ human fibroblasts treated with DMSO (*left*) itraconazole (*middle*) and posaconazole (*right*) for 72 h. Drugs were then removed and chased for 0 (*top*) or 48 (*bottom*) hours before staining with filipin. Scale bar = 10 μm.

Given that the half-life of wild-type NPC1 is about 42 h (5, 12), we hypothesized that correctly folded NPC1 protein might persist and remain functional in the lysosomes after drug removal. To test this hypothesis, we evaluated the cholesterol storage phenotype after drug removal with 4 different doses and time points. *NPC1*^*I1061T/I1061T*^ human fibroblasts were treated with itraconazole or posaconazole at various concentrations for 72 h. After treatment, the cells were rinsed and fixed immediately or incubated in fresh growth medium without drugs for an additional 24, 48, or 72 h. The initial 72-h treatment with either drug did not reduce lysosomal cholesterol accumulation compared to DMSO controls (Fig. 2, *A* and *B*). In fact, treatment with 0.33 μM itraconazole resulted in slightly increased cholesterol accumulation (Fig. 2*A*). However, following drug removal, both drugs showed a significant decrease in lysosomal cholesterol accumulation after 24 and 48 h of washout (Fig. 2, *A* and *B*). This effect was also observed in *NPC1*^*N1156S/R1186H*^ human fibroblasts after 48 h of washout at 0.33 μM (Fig. S1). By 72 h of washout, a partial rebound in cholesterol levels was observed, likely due to the degradation of NPC1 proteins over time (Fig. 2, *A* and *B*).

To further evaluate the balance between the chaperone and inhibitory effects of itraconazole and posaconazole, we employed a repeated pulse dosing treatment in which *NPC1*^*I1061T/I1061T*^ human fibroblasts were treated with 0.33 μM of either compound for 6 h, followed by an 18-h washout in fresh medium. This cycle was repeated daily for 4 days. At the end of the treatment period, Filipin staining revealed a significant reduction in lysosomal cholesterol accumulation compared to DMSO controls (Fig. 2*C*). Both strategies preserved the chaperone activity of the drugs by promoting proper folding of NPC1, while mitigating their inhibitory effects by prolonged or intermittent washout.

### Itraconazole and posaconazole correct NPC1 localization in *NPC1*^*I1061T/I1061T*^ human fibroblasts

**Figure 3:**
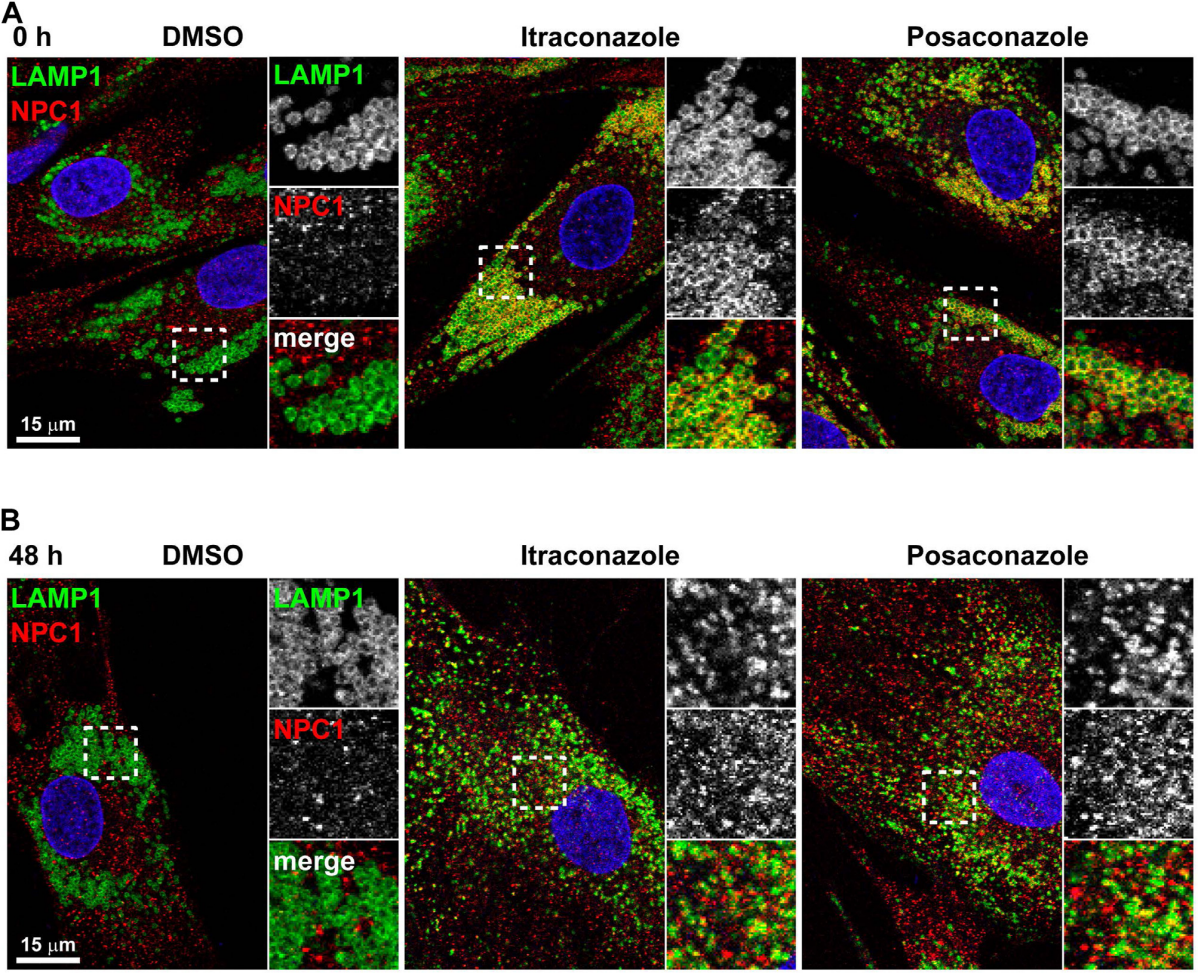
**Localization of NPC1 in itraconazole or posaconazole-treated *NPC1*^*I1061T/I1061T*^ human fibroblasts**. Confocal images of fixed *NPC1*^*I1061T/I1061T*^ human fibroblasts. Cells were treated with DMSO (*left*), itraconazole (*middle*), or posaconazole (*right*) at a concentration of 0.33 μM for 72 h and chased for 0 (*A*) or 48 (*B*) hours without drugs. Cells were then fixed and stained with mouse anti-LAMP1 (*green*) and rabbit anti-NPC1 (*red*) primary antibodies followed by anti-mouse AlexaFluor-488 and anti-rabbit AlexaFluor-568 secondary antibodies. *Dashed boxes* mark regions magnified 2.56-fold in insets.

To confirm the chaperone effects of itraconazole and posaconazole, we examined the localization of NPC1 on LE/Ly in *NPC1*^*I1061T/I1061T*^ human fibroblasts. After 72 h of treatment, NPC1 was successfully trafficked to LAMP1-positive LE/Ly, in contrast to DMSO-treated controls where NPC1 trafficking was impaired (Fig. 3*A*). Interestingly, these LE/Ly appeared enlarged, hollow, and clustered around the perinuclear regions, consistent with lysosomal dysfunction and impaired cholesterol export (Fig. 3*A*). Remarkably, after a 48-h drug washout, NPC1 localization to LAMP1-positive compartments persisted, but the LE/Ly were no longer hollow and no longer clustered in the perinuclear region, suggesting they were functional (Fig. 3*B*), indicating a restoration of lysosomal function. These immunofluorescence data provided further evidence that folded NPC1 remained functional in LE/Ly after drug removal.

## Discussion

In this study, we confirmed inhibitory effects of itraconazole and posaconazole on NPC1-mediated cholesterol transport out of lysosomes in normal human fibroblasts (Fig. 1), consistent with a previous report (19). We further demonstrated that these compounds promote lysosomal localization of mutant NPC1 proteins both when present in the media and after washout (Fig. 3), indicating improved folding and trafficking. Although lysosomal dysfunction was observed during treatment, our findings show that these inhibitory effects are reversible. Following drug removal, lysosomal cholesterol levels were reduced significantly in *NPC1*^*I1061T/I1061T*^ human fibroblasts after 24 and 48 h of washout at 0.11 to 0.33 μM (Fig. 2, *A* and *B*). A similar effect was also observed in *NPC1*^*N1156S/R1186H*^ human fibroblasts after 48 h of washout (Fig. S1). These results suggest that properly folded NPC1 proteins remain functional in lysosomes after the compounds are removed. However, the gradual loss of effect over time is likely due to degradation of NPC1 proteins in the lysosomes (Fig. 2, *A* and *B*). These results align with previous reports that wild-type NPC1 proteins have a half-life of about 42 h (5, 12) allowing sufficient time for activity after the drug is cleared.

A key aspect of our study was the use of repeated pulse dosing of itraconazole and posaconazole in cultured cells. In clinical settings, drugs are typically taken once or twice daily, resulting in plasma concentration peaks followed by metabolic clearance. By repeating short-duration drug pulses followed by extended washout periods, we observed a significant reduction in lysosomal cholesterol accumulation compared to DMSO controls (Fig. 2*C*). This strategy mimics the possible pharmacokinetics of the drug in patients, where folded NPC1 can accumulate during treatment and function during drug-free intervals, which provides a clinically relevant strategy to evaluate the therapeutic potential of reversible inhibitors that also act as pharmacological chaperones.

The challenges associated with NPC1^I1061T^ misfolding and trafficking defects share remarkable parallels with other disorders involving mutant proteins, such as cystic fibrosis caused by defective cystic fibrosis transmembrane conductance regulator (CFTR). In cystic fibrosis, the common F508del mutation results in a misfolded CFTR protein retained and targeted for degradation in the ER, preventing it from reaching its functional site at the plasma membrane (20). Small molecules like lumacaftor and tezacaftor have been successfully developed to stabilize this mutant protein, enabling its trafficking and partial restoration of function (20, 21).

The success of migalastat in treating Fabry disease offers a precedent for using pharmacological chaperones with inhibitory effects to stabilize mutant proteins and restore function (22, 23). Migalastat binds to mutant α-galactosidase A, enhancing its proper folding, trafficking, and enzymatic activity, ultimately mitigating Fabry disease symptoms. However, at high doses, migalastat can inhibit α-galactosidase A. Similarly, the iminosugar isofagomine, a reversible active site inhibitor of lysosomal acid β-glucosidase, allows the mutant enzyme to be transported to the lysosome, where it is functional after washout (24). These examples highlight the therapeutic potential of reversible inhibitory pharmacological chaperones in managing protein misfolding disorders, such as NPC disease, while emphasizing the critical need to optimize treatment strategies to balance chaperone benefits with inhibitory effects.

Our findings have important implications for the potential therapeutic application of small molecules that bind mutant NPC1 proteins in the treatment of NPC disease. Itraconazole and posaconazole are examples of small molecules that can act as chaperones to assist in the folding of misfolded mutant NPC1 proteins. However, their dual chaperone/inhibitory effects present significant challenges for clinical use. Additionally, they have poor penetration of the central nervous system (25, 26) and numerous side effects and drug interactions. Nevertheless, the washout approach employed in this study offers a potential strategy to mitigate the inhibitory effects while preserving the chaperone benefits. Further work will be required to identify nontoxic molecules that bind reversibly and promote proper folding without compromising lysosomal function.

## Experimental procedures

### Reagents

MEM (ThermoFisher #11095098). Fetal Bovine Serum (GeminiBio #100–106–500). Saponin (Calbiochem #558255). Rabbit-anti NPC1 (Abcam #ab134113). Mouse-anti LAMP1 (Cell Signaling #15665S). Goat anti-mouse AlexaFluor-488 (Thermo Fisher #A11001). Goat anti-rabbit AlexaFluor-568 (Thermo Fisher # A11011). Hoechst 33,342 (Cayman Chemical #15547). PFA (Electron Microscopy Sciences #15714-S). Filipin (Sigma #F9765). Draq5 (Thermo Fisher #62251). NucSpot 650/665 (Biotium #41034). DMSO (Sigma # 34869–100 ml). Itraconazole (Cayman Chemical #13288). Posaconazole (Cayman Chemical #14737–1).

### Cell culture and media

Normal human fibroblasts (GM05659), *NPC1*^*I1061T/I1061T*^ human fibroblasts (GM18453), and *NPC1*^*N1156S/R1186H*^ human fibroblasts (GM18397) were obtained from the Coriell Institute. Cells were maintained in MEM supplemented with 10% FBS. For drug treatments, cells were cultured in MEM supplemented with 5% FBS.

### Inhibitory effects of itraconazole and posaconazole

Normal human fibroblasts were seeded in 96-well plates (Corning #3904) with 1600 cells/well. After overnight incubation, 2 × concentrated compounds were added to each well at a final concentration of 0.33 μM for 72 h. At the end of the treatment, cells were washed 3 times with PBS, fixed with 1.5% PFA for 20 min, and washed again 3 times with PBS. Cells were then stained with 50 μg/ml filipin for 45 min, washed 3 additional times, and counterstained with 1.5 μM Draq5 nuclear stain.

### Time and dose dependence under continuous and repeated pulsed drug treatment

#### Continuous treatment assay

The time and dose dependence of itraconazole and posaconazole was examined in *NPC1*^*I1061T/I1061T*^ human fibroblasts after 72 h of treatment and followed by incubation for an additional 0, 24, 48, or 72 h in the absence of drugs. The assay is based on the use of filipin, a fluorescent dye that binds to unesterified cholesterol. Cells were seeded in 96-well plates (Corning #3904) at 3 different densities, with 1600 cells/well for 72-h treatments, 900 cells/well for 72-h treatments followed by 24- or 48-h chase, and 500 cells/well for 72-h treatments followed by a 72-h chase. After overnight incubation, 2 × concentrated drugs in drug treatment media were added at 4 different doses, such that the final concentration ranged from 12 nM to 0.33 μM. DMSO was used as a control for each concentration. At the end of each time point, cells were washed 3 times with PBS, fixed with 1.5% PFA for 20 min, and washed again 3 times with PBS. Cells were then stained with 50 μg/ml filipin for 45 min, washed 3 additional times, and counterstained with 1.5 μM Draq5 nuclear stain.

#### Repeated pulse dosing treatment assay

*NPC1*^*I1061T/I1061T*^ human fibroblasts were seeded in 96-well plates (Corning #3904) with 330 cells/well. After 3 days of incubation, 2 × concentrated compounds were added to each well at a final concentration of 0.33 μM for 6 h, followed by an overnight washout in fresh medium (MEM supplemented with 5% FBS) for 18 h, and this cycle was repeated for 4 days. At the end of the treatment, cells were washed 3 times with PBS, fixed with 1.5% PFA for 20 min, and washed again 3 times with PBS. Cells were then stained with 50 μg/ml filipin and 1:20,000 NucSpot 650/665 (Biotium #41034) for 45 min, followed by 3 additional PBS washes.

### Immunolocalization of NPC1

*NPC1*^*I1061T/I1061T*^ human fibroblasts were seeded at two different densities in 96-well plates with glass-like polymer coverslip bottoms (Cellvis #P96–1.5P), with 1500 cells/well for 72-h treatments and 900 cells/well for 72-h treatments followed by a 48-h chase. After overnight incubation, at the end of each time point, cells were fixed with 1.5% PFA followed by blocking and permeabilization with 10% normal goat serum and 0.1% saponin in PBS for 1h at room temperature. Rabbit-anti NPC1 (1:1000) and mouse-anti LAMP1 (1:1000) primary antibodies were diluted in blocking buffer and incubated overnight at 4 °C. Goat anti-mouse AlexaFluor-488 (1:500) and Goat anti-rabbit AlexaFluor-568 (1:500) secondary antibodies were diluted in PBS with 0.1% saponin and incubated for 40 min at room temperature. 2 μg/ml Hoechst 33,342 was incubated alongside secondary antibodies to stain nuclei.

### Fluorescence microscopy

For Filipin intensity quantifications, images were acquired on an ImageXpressMicro automatic fluorescence microscope with a 10x air objective (NA 0.45). Nine fields of view were imaged in each well (200–600 cells), and lysosome-like storage organelle (LSO) analysis was performed.

High-resolution Filipin micrographs were acquired on a Zeiss Axiovert7 fluorescence microscope with a 63x oil objective (NA 1.4) and a Zeiss Axiocam 705 mono R2 camera.

For immunofluorescence microscopy of Hoechst, NPC1, and LAMP1, images were acquired on a Leica Stellaris eight confocal microscope with a 63x oil objective (NA 1.4).

### LSO analysis

Measurements were made from nine fields of view, approximately 200 to 600 cells per field, with two wells per condition in each experiment, and the experiment was repeated 3 times. Images were analyzed to obtain the LSO ratio, representing the filipin fluorescence intensity in the brightly labeled organelles in the field divided by the total area of the cells (11). The LSO ratio is determined using two thresholds that are applied to the Filipin images. A low threshold is set to include all areas occupied by cells, aligning with cell outlines visible in transmitted light images. A higher threshold is then set to identify regions brightly stained with filipin in cells. The thresholds were chosen for each experiment.

LSO ratio = total intensity above high–thresholded filipin intensity/number of pixels above low – low-low-thresholded filipin intensity.

A high LSO ratio is associated with high levels of cholesterol in late endosomes and lysosomes. Removal of this cholesterol leads to a reduction in the LSO ratio. The LSO ratio for each concentration was normalized to the corresponding DMSO-treated control.

### Statistical analysis

The effect of itraconazole and posaconazole on normal human fibroblasts was analyzed using one-way ANOVA (Brown-Forsythe and Welch ANOVA tests) with multiple comparisons against DMSO controls using GraphPad Prism 10. ∗∗∗, *p* ≤ 0.001; ∗∗∗∗, *p* ≤ 0.0001.

For time and dose-dependent effects of itraconazole and posaconazole on *NPC1*^*I1061T/I1061T*^ human fibroblasts, continuous treatment assay data were analyzed using Two-Way ANOVA with multiple comparison against 72 h of drug incubation using GraphPad Prism 10. Different colors were assigned to represent different time points. ∗, *p* ≤ 0.05; ∗∗, *p* ≤ 0.01; ∗∗∗, *p* ≤ 0.001; ∗∗∗∗, *p* ≤ 0.0001; ns, not significant.

For the repeated pulse dosing treatment assay, data were analyzed using One-Way ANOVA (Brown-Forsythe and Welch ANOVA tests) with multiple comparisons against DMSO controls using GraphPad Prism 10. ∗∗∗∗, *p* ≤ 0.0001.

## Data availability

The authors declare that the data underlying the findings of this study are available within the article and its Supporting Information and are available upon request.

## Supporting information

This article contains supporting information.

## Conflict of interest

The authors declare that they have no conflict of interest with the contents of this article.

## References

[1] Rosenbaum A.I., Maxfield F.R. (2011). Niemann-Pick type C disease: molecular mechanisms and potential therapeutic approaches. J. Neurochem..

[2] Vanier M.T., Millat G. (2003). Niemann-Pick disease type C. Clin. Genet..

[3] Dardis A., Zampieri S., Gellera C., Carrozzo R., Cattarossi S., Peruzzo P. (2020). Molecular genetics of niemann-pick type C disease in Italy: an update on 105 patients and description of 18 NPC1 novel variants. J. Clin. Med..

[4] Millat G., Marcais C., Rafi M.A., Yamamoto T., Morris J.A., Pentchev P.G. (1999). Niemann-Pick C1 disease: the I1061T substitution is a frequent mutant allele in patients of Western European descent and correlates with a classic juvenile phenotype. Am. J. Hum. Genet..

[5] Gelsthorpe M.E., Baumann N., Millard E., Gale S.E., Langmade S.J., Schaffer J.E. (2008). Niemann-Pick type C1 I1061T mutant encodes a functional protein that is selected for endoplasmic reticulum-associated degradation due to protein misfolding. J. Biol. Chem..

[6] Rosenbaum A.I., Zhang G.T., Warren J.D., Maxfield F.R. (2010). Endocytosis of beta-cyclodextrins is responsible for cholesterol reduction in Niemann-Pick type C mutant cells. Proc. Natl. Acad. Sci. U. S. A..

[7] Davidson C.D., Ali N.F., Micsenyi M.C., Stephney G., Renault S., Dobrenis K. (2009). Chronic cyclodextrin treatment of murine Niemann-Pick C disease ameliorates neuronal cholesterol and glycosphingolipid storage and disease progression. PLoS One.

[8] Liu B., Li H., Repa J.J., Turley S.D., Dietschy J.M. (2008). Genetic variations and treatments that affect the lifespan of the NPC1 mouse. J. Lipid. Res..

[9] Vite C.H., Bagel J.H., Swain G.P., Prociuk M., Sikora T.U., Stein V.M. (2015). Intracisternal cyclodextrin prevents cerebellar dysfunction and Purkinje cell death in feline Niemann-Pick type C1 disease. Sci. Transl. Med..

[10] Mengel E., Patterson M.C., Da Riol R.M., Del Toro M., Deodato F., Gautschi M. (2021). Efficacy and safety of arimoclomol in Niemann-Pick disease type C: results from a double-blind, randomised, placebo-controlled, multinational phase 2/3 trial of a novel treatment. J. Inherit. Metab. Dis..

[11] Pipalia N.H., Saad S.Z., Subramanian K., Cross A., Al-Motawa A., Garg K. (2021). HSP90 inhibitors reduce cholesterol storage in Niemann-Pick type C1 mutant fibroblasts. J. Lipid. Res..

[12] Pipalia N.H., Subramanian K., Mao S., Ralph H., Hutt D.M., Scott S.M. (2017). Histone deacetylase inhibitors correct the cholesterol storage defect in most Niemann-Pick C1 mutant cells. J. Lipid. Res..

[13] Pipalia N.H., Cosner C.C., Huang A., Chatterjee A., Bourbon P., Farley N. (2011). Histone deacetylase inhibitor treatment dramatically reduces cholesterol accumulation in Niemann-Pick type C1 mutant human fibroblasts. Proc. Natl. Acad. Sci. U. S. A..

[14] Cruz D.L., Pipalia N., Mao S., Gadi D., Liu G., Grigalunas M. (2021). Inhibition of histone deacetylases 1, 2, and 3 enhances clearance of cholesterol accumulation in niemann-pick C1 fibroblasts. ACS. Pharmacol. Transl. Sci..

[15] Ohgane K., Karaki F., Dodo K., Hashimoto Y. (2013). Discovery of oxysterol-derived pharmacological chaperones for NPC1: implication for the existence of second sterol-binding site. Chem. Biol..

[16] Shioi R., Karaki F., Yoshioka H., Noguchi-Yachide T., Ishikawa M., Dodo K. (2020). Image-based screen capturing misfolding status of Niemann-Pick type C1 identifies potential candidates for chaperone drugs. PLoS One.

[17] Nagappan V., Deresinski S. (2007). Reviews of anti-infective agents: posaconazole: a broad-spectrum triazole antifungal agent. Clin. Infect. Dis..

[18] Peyton L.R., Gallagher S., Hashemzadeh M. (2015). Triazole antifungals: a review. Drugs. Today. (Barc).

[19] Trinh M.N., Lu F., Li X., Das A., Liang Q., De Brabander J.K. (2017). Triazoles inhibit cholesterol export from lysosomes by binding to NPC1. Proc. Natl. Acad. Sci. U. S. A..

[20] Brown C.R., Hong-Brown L.Q., Biwersi J., Verkman A.S., Welch W.J. (1996). Chemical chaperones correct the mutant phenotype of the delta F508 cystic fibrosis transmembrane conductance regulator protein. Cell. Stress. Chaperones..

[21] McDonald E.F., Sabusap C.M.P., Kim M., Plate L. (2022). Distinct proteostasis states drive pharmacologic chaperone susceptibility for cystic fibrosis transmembrane conductance regulator misfolding mutants. Mol. Biol. Cell..

[22] Asano N., Ishii S., Kizu H., Ikeda K., Yasuda K., Kato A. (2000). In vitro inhibition and intracellular enhancement of lysosomal alpha-galactosidase A activity in Fabry lymphoblasts by 1-deoxygalactonojirimycin and its derivatives. Eur. J. Biochem..

[23] Fan J.Q., Ishii S. (2007). Active-site-specific chaperone therapy for Fabry disease. Yin and Yang of enzyme inhibitors. FEBS J..

[24] Steet R.A., Chung S., Wustman B., Powe A., Do H., Kornfeld S.A. (2006). The iminosugar isofagomine increases the activity of N370S mutant acid β-glucosidase in Gaucher fibroblasts by several mechanisms. Proc. Natl. Acad. Sci. U. S. A..

[25] Wiederhold N.P., Pennick G.J., Dorsey S.A., Furmaga W., Lewis J.S., Patterson T.F. (2014). A reference laboratory experience of clinically achievable voriconazole, posaconazole, and itraconazole concentrations within the bloodstream and cerebral spinal fluid. Antimicrob. Agents. Chemother..

[26] Barde F., Billaud E., Goldwirt L., Horodyckid C., Jullien V., Lanternier F. (2019). Low central nervous system posaconazole concentrations during cerebral phaeohyphomycosis. Antimicrob. Agents. Chemother..

