# Supplementary material for: Itraconazole and posaconazole, inhibitors of NPC1 sterol transport, act as pharmacological chaperones after washout
Source: J Biol Chem. 2025 Jun 16;301(7):110370. doi: 10.1016/j.jbc.2025.110370 (PMC12272866; doi:10.1016/j.jbc.2025.110370)
Supplement: Supplementary Material [file mmc1.docx]

**Itraconazole and Posaconazole, Inhibitors of NPC1 Sterol Transport, Act as Pharmacological Chaperones After Washout**

Weixiang A. Wang^1,^ *, Cheng-I J. Ma^1,^ *, Noah Steinfeld^1^, Frederick R. Maxfield^1,†^

^1^Department of Biochemistry, Weill Cornell Medicine, New York, NY, USA

*These authors contributed equally to this work.

^†^Correspondence

Frederick R. Maxfield

[frmaxfie@med.cornell.edu](mailto:frmaxfie@med.cornell.edu)

Supporting information – Methods, Figure and Figure Legend

**Methods**

**Effects of itraconazole and posaconazole on *NPC1^N1156S/R1186H^* human fibroblasts.**

*NPC1^N1156S/R1186H^* human fibroblasts were seeded in 96-well plates (Corning #3904) at two different densities, with 1800 cells/well for 72-hour treatments, 1100 cells/well for 72-hour treatments followed by 48-hour chase. After overnight incubation, 2× concentrated drugs in drug treatment media were added to achieve a final drug concentration of 0.33 μM. DMSO was used as a control. At the end of each time point, cells were washed three times with PBS, fixed with 1.5% PFA for 20 minutes, and washed again three times with PBS. Cells were then stained with 50 µg/mL filipin and 1:20,000 NucSpot 650/665 (Biotium #41034) for 45 minutes, followed by three additional PBS washes.


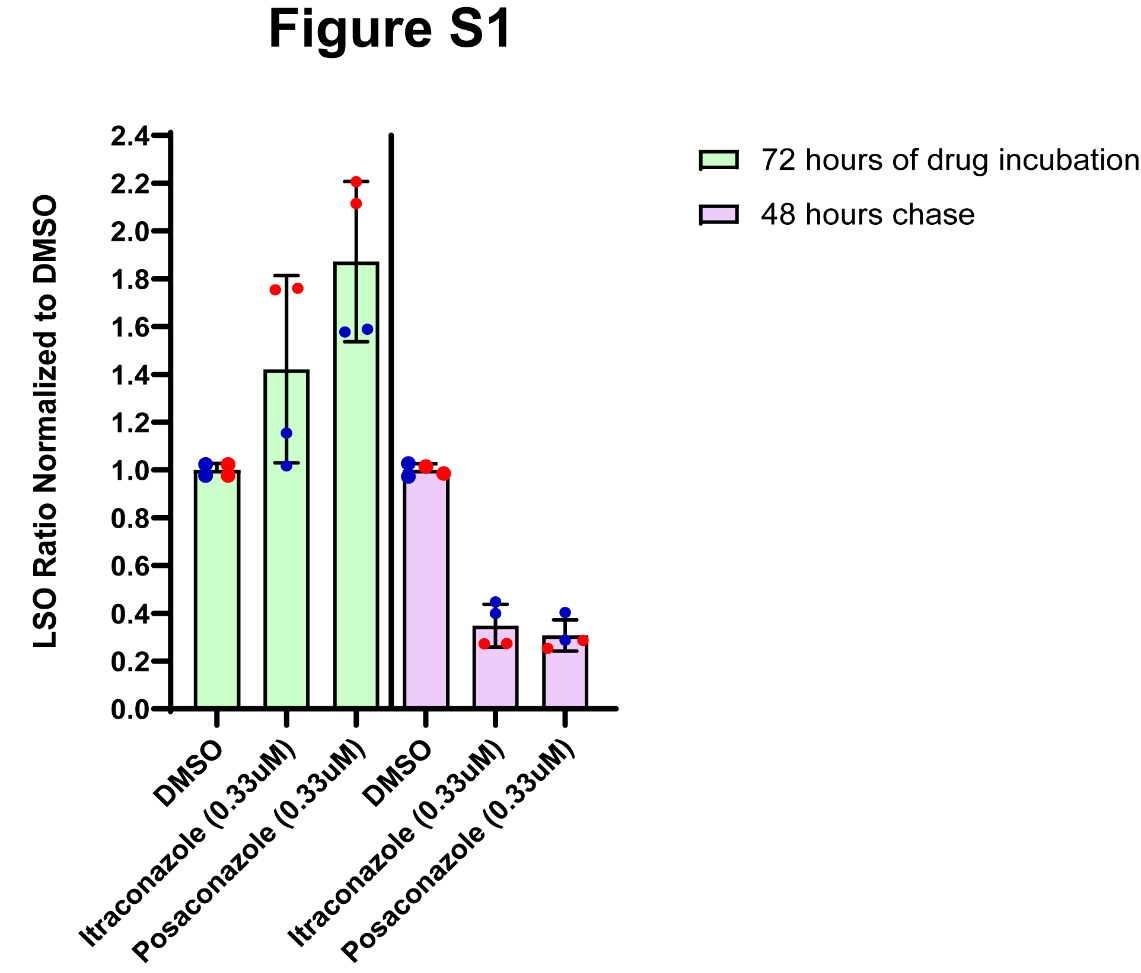


**Figure S1. Effects of itraconazole and posaconazole on *NPC1^N1156S/R1186H^* human fibroblasts.**

*NPC1^N1156S/R1186H^* human fibroblasts were treated with itraconazole or posaconazole at a final concentration of 0.33 µM for 72 hours and chased for an additional 0 or 48 hours without drugs. At the end of each time point, cells were fixed and stained with filipin and NucSpot 650/665 for 45 min followed by three additional PBS washes.The LSO per cell values were measured to determine the relative amount of stored cholesterol in LE/Ly. Data represent two independent experiments, each shown in a different color. Each data point was obtained by using 9 field of views in one well. Each field of view contains about 50-250 cells. Green bars indicate 72 hours of drug treatment; purple bars indicate 72 hours of drug treatment following by 48 hours washout. LSO values in DMSO-treated cells were measured in each experiment for normalization. The LSO value of one indicates the value in DMSO controls. Error bars show standard deviation.
